# Bioinformatics analysis of genes related to iron death in diabetic nephropathy through network and pathway levels based approaches

**DOI:** 10.1371/journal.pone.0259436

**Published:** 2021-11-04

**Authors:** Yaling Hu, Shuang Liu, Wenyuan Liu, Ziyuan Zhang, Yuxiang Liu, Dalin Sun, Mingyu Zhang, Jingai Fang

**Affiliations:** 1 Shanxi Medical University, Taiyuan, Shanxi, China; 2 Department of Nephrology, The First Hospital of Shanxi Medical University, Taiyuan, Shanxi, China; 3 Department of Urology, The First Hospital of Shanxi Medical University, Taiyuan, Shanxi, China; University of Science and Technology Liaoning, CHINA

## Abstract

Diabetic nephropathy is one of the common microvascular complications of diabetes. Iron death is a recently reported way of cell death. To explore the effects of iron death on diabetic nephropathy, iron death score of diabetic nephropathy was analyzed based on the network and pathway levels. Furthermore, markers related to iron death were screened. Using RNA-seq data of diabetic nephropathy, samples were clustered uniformly and the disease was classified. Differentially expressed gene analysis was conducted on the typed disease samples, and the WGCNA algorithm was used to obtain key modules. String database was used to perform protein interaction analysis on key module genes for the selection of Hub genes. Moreover, principal component analysis method was applied to get transcription factors and non-coding genes, which interact with the Hub gene. All samples can be divided into two categories and principal component analysis shows that the two categories are significantly different. Hub genes (FPR3, C3AR1, CD14, ITGB2, RAC2 and ITGAM) related to iron death in diabetic nephropathy were obtained through gene expression differential analysis between different subtypes. Non-coding genes that interact with Hub genes, including hsa-miR-572, hsa-miR-29a-3p, hsa-miR-29b-3p, hsa-miR-208a-3p, hsa-miR-153-3p and hsa-miR-29c-3p, may be related to diabetic nephropathy. Transcription factors HIF1α, KLF4, KLF5, RUNX1, SP1, VDR and WT1 may be related to diabetic nephropathy. The above factors and Hub genes are collectively involved in the occurrence and development of diabetic nephropathy, which can be further studied in the future. Moreover, these factors and genes may be potential target for therapeutic drugs.

## Introduction

Diabetic nephropathy is one of the most common microvascular complications of diabetes [1]. Once it develops to end-stage renal disease treatment is difficult, thus active and early prevention and treatment are of great significance to delay the progression of the disease. The etiology and pathogenesis of diabetic nephropathy are still unclear. It is a multifactorially induced disease with a combination of genetic background and some risk factors [2].

Iron homeostasis is an important factor in maintaining the normal function of kidney cells [3]. Excessive iron deposition in kidney cells can cause tissue dysfunction [4]. The abnormal accumulation of iron may promote the occurrence and development of diabetic nephropathy by increasing oxidative/nutritional stress and reducing antioxidant capacity [5, 6]. The discovery of iron death provides a new explanation for the role of iron overload in the pathogenesis of the disease.

Iron death is a new type of programmed cell death characterized by iron-dependent lipid hydrogen peroxide accumulation and loss of lipid repair enzyme Glutathione peroxidase 4 (GPX4) activity [7]. When iron death occurs, the low expression of ferritin 1 (Transferrin receptor-1, FTH-1) and the overexpression of transferrin receptor 1 (Transferrin Receptor-1, TFR-1) lead to excessive accumulation of ferrous ions [8]. The fenton reaction promotes the production of a large number of reactive oxygen species (ROS) [8]. Meanwhile, the cystine/glutamate reverse transport system (Xc-system) function is destroyed, leading to the consumption of glutathione produced by the body and preventing GPX4 from exerting its normal antioxidant capacity [9], which finally makes cell membranes, containing phospholipids, extremely vulnerable to ROS attack. The final products of lipid peroxidation are malondialdehyde (MDA) and 4-hydroxynonenal (4-HNE) accumulate, which eventually leads to significant cytotoxicity and induces cell iron death [10].

Studies have shown that diabetic nephropathy may be related to iron death. Animal studies have found that [11], diabetic nephropathy models possess characteristic indications of iron death, such as weakened antioxidant capacity, iron overload, and lipid peroxidation product accumulation. Moreover, inhibition of iron death can delay the development of kidney pathology in diabetic mice. Experiments *in vitro* have shown that both the iron death activator Erastin and high glucose can induce the iron death of glomerular mesangial cells [12]. The induction of iron death is related to the up-regulation of long-chain acyl-CoA synthetase-4 (ACSL4), prostaglandin-endoperoxide synthas 2 (PTGS2), NADPH oxidase 1 (Nicotinamide Adenine Dinucleotide Phosphate Oxidase 1, NOX1) and the down-regulation of GPX4 [13].

In patients with diabetic nephropathy, the iron death-related factors ACSL4, PTGS2, and NOX1 were significantly increased, and GPX4 was significantly decreased [12]. The iron death marker ACSL4 is mainly expressed in the renal tubules. Wang et al. found that the knockout or overexpression of ACSL4 gene caused changes in the sensitivity of renal tubular epithelial cells to iron death [14]. Furthermore, ACSL4 inhibitors can block the iron death of renal tubular cells and inhibit the production of pro-inflammatory cytokines, which finally relieves the symptoms of diabetic nephropathy [14]. In renal tubular epithelial cells treated with TGF-β1, the characteristics of iron death are obvious. A significant decrease in glutathione levels and expression of cystine/glutamate reverse transport solute carriers (SLC7A11) and GPX4 are observed [15]. Meanwhile, the lipid peroxidation is significantly increased and iron death inhibitors can significantly improve TGF-β1-induced renal tubular epithelial cell death [15]. The above studies suggest that iron death plays an important role in the occurrence and development of diabetic nephropathy, but the underlying mechanism is still unclear.

Recent studies have used bioinformatics methods to identify genes related to iron death. These studies can help identify potential disease-related key genes [16], predict disease prognosis models [17], discover potential biomarkers and therapeutic targets [18], and provide new strategies for individualized treatment. Liang et al. found that most genes related to iron death are differentially expressed between liver cancer and adjacent normal tissues, and iron death-related gene markers can be used to predict the prognosis of liver cancer [19]. Zhu et al. found that iron death-related genes that may be involved in esophageal adenocarcinoma have important value in predicting osteosarcoma [20].

**Fig 1:**
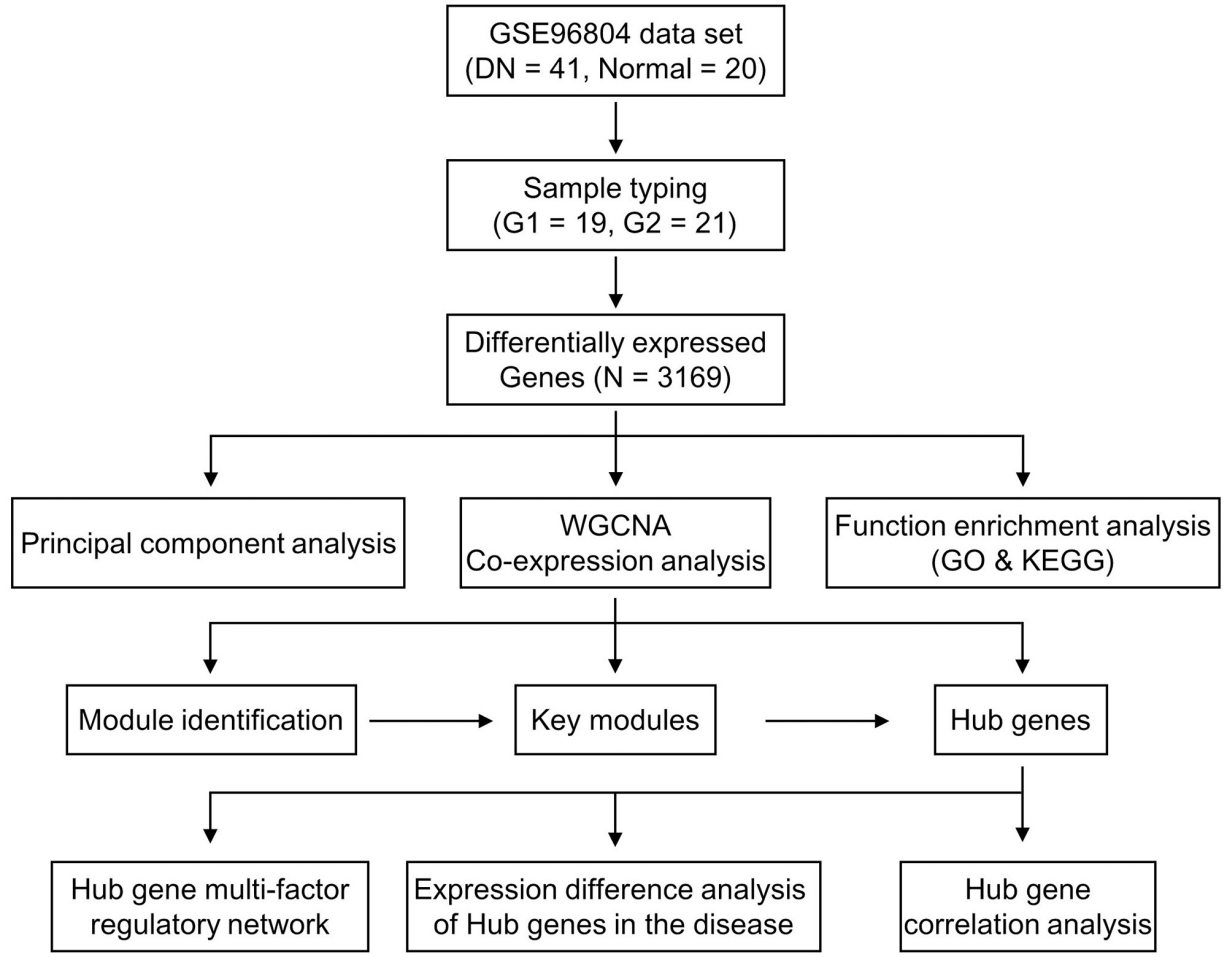
Flow chart of the bioinformatics analysis.

Based on the above research, in order to explore the influence of iron death factor on diabetic nephropathy, this study clustered the diseases based on iron death factor to obtain different disease subtypes. Based on the analysis of differentially expressed genes between different subtypes, the key modules of the disease were screened, and finally the key genes related to iron death in diabetic nephropathy were obtained. Further, a multi-factor regulatory network for key genes was constructed and their regulatory relationship with transcription factors (TF) and non-coding RNA (ncRNA) was also analyzed. The flow chart of bioinformatics analysis of this article was presented in Fig 1. This study provides a basis information for exploring the potential molecular mechanisms in the development of diabetic nephropathy.

## Results

### Diabetic nephropathy dataset and iron death factor

RNA-seq data GSE96804 was selected as the gene expression data related to diabetic nephropathy. This dataset includes 41 diabetic nephropathy patients and 20 normal control patients, from which the transcriptome between the glomerulus and the glomerulus not affected by tumor nephrectomy is compared (Table 1). Ferroptosis genes (60 genes, summarized in Table 2) were selected from previous report [19].

**Table 1:** RNA-seq expression profile data set from GEO database.

DatasetID	Platform	Test	Control
**GSE96804**	GPL17586	41	20

**Table 2:** Iron death factors.

Ferrotosis-related genes	Name
*ACSL4*	acyl-CoA synthetase long-chain family member 4
*AKR1C1*	aldo-keto reductase family 1 member C1
*AKR1C2*	aldo-keto reductase family 1 member C2
*AKR1C3*	aldo-keto reductase family 1 member C3
*ALOX15*	arachidonate 15-lipoxygenase
*ALOX5*	arachidonate 5-lipoxygenase
*ALOX12*	arachidonate 12-lipoxygenase
*ATP5MC3*	ATP synthase membrane subunit c locus 3
*CARS*	cysteinyl tRNA synthetase
*CBS*	cystathion ine beta synthase
*CD44*	CD44 molecule
*CHAC1*	ChaC glutathione- specific gamma-glutamyl cyclotransferase 1
*CISD1*	CDGSH iron sulfur domain 1
*CS*	citrate synthase
*DPP4*	dipeptidyl-dippeptidase-4
*FANCD2*	fanconi anemia comple mentation group D2
*GCLC*	glutamate-cysteine ligase catalytic subunit
*GCLM*	glutamate-cysteine ligase modifier subunit
*GLS2*	glutaminase 2
*GPX4*	glutathio ne peroxidase 4
*GSS*	glutathione synthetase
*HMGCR*	3-hydroxy-3- methylglutaryl-CoA reductase
*HSPB1*	heat shock protein beta 1
*CRYAB*	heat shock protein beta 5
*LPCAT3*	lysophosp hatidylcholine acyltransferase 3
*MT1G*	metallothionein-1G
*NCOA4*	nuclear receptor coactiva tor 4
*PTGS2*	prostagla ndin-endoperoxide synthase 2
*RPL8*	ribosomal protein L8
*SAT1*	spermidine/spermine N1-acetyltra nsferase 1
*SLC7A11*	solute carrier family 7 member 11
*FDFT1*	farnesyl-diphosphate farnesyltransferase 1
*TFRC*	transferrin receptor
*TP53*	tumor protein 53
*EMC2*	ER membrane protein complex subunit 2
*AIFM2*	apoptosis inducing factor mitochondria associated 2
*PHKG2*	phospho rylase kinase, g2
*HSBP1*	heat-shock 27-k Da protein 1
*ACO1*	aconitase 1
*FTH1*	ferritin heavy chain 1
*STEAP3*	six-transm embrane epithelial antigen of prostate 3
*NFS1*	cysteine desulfurase
*ACSL3*	acyl-CoA synthetase long-chain family member 3
*ACACA*	acetyl-CoA carboxylase alpha
*PEBP1*	phosphatidy lethanolamine-binding protein 1
*ZEB1*	zinc finger E-box-binding homeobox 1
*SQLE*	squalene monooxygenase
*FADS2*	fatty acid desaturase 2/acyl-CoA 6-desaturase
*NFE2L2*	nuclear factor, erythroid 2 like 2
*KEAP1*	kelch-like ECH- associated protein 1
*NQO1*	quinone oxidoreductas e-1
*NOX1*	NADPH oxidase 1
*ABCC1*	ATP binding cassette subfamily C member 1
*SLC1A5*	solute carrier family 1 member 5
*GOT1*	glutamic-oxa loacetic transaminase 1
*G6PD*	glucose-6-phosphate dehydrogenas e
*PGD*	phosphoglycerate dehydrogenas e
*IREB2*	iron response element-binding protein 2
*HMOX1*	heme oxygenase 1
*ACSF2*	acyl-CoA synthetase family member 2

### The expression distribution of iron death factors in diabetic nephropathy dataset

**Fig 2:**
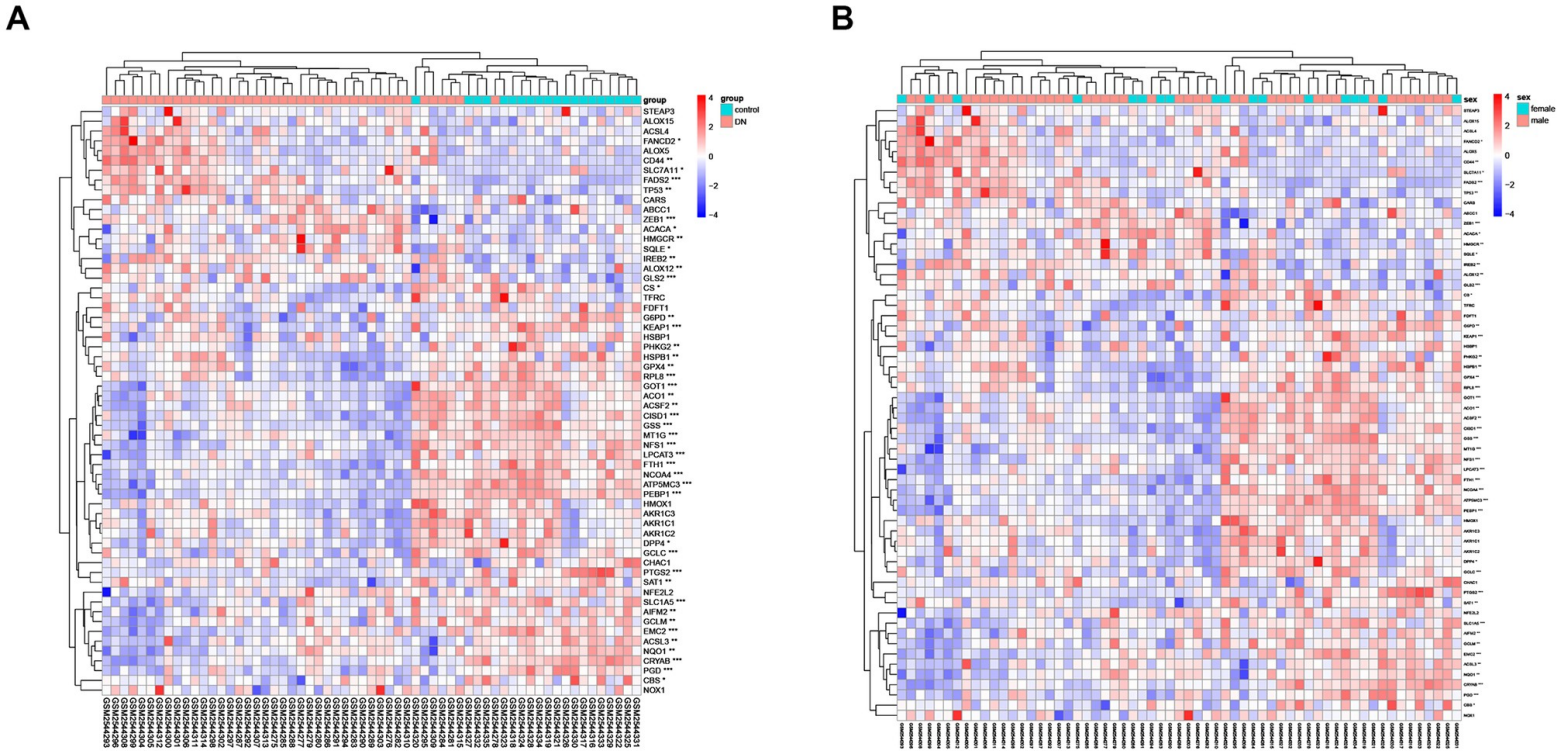
Heat maps of iron death factor expression clustered by sample type (A) and gender (B).

Extracting the iron death gene expression data from the diabetic nephropathy-related gene expression data set and analyzing the differential expression according to the sample type (diabetic nephropathy group and normal control group) and gender (male and female). Analyzed by sample types, there were 44 iron death genes in diabetic nephropathy group and normal control group with expression differences (p<0.05) (Fig 2A). Analyzed by genders, no difference in the expression of iron death gene was observed between men and women (Fig 2B).

### Types of diabetic nephropathy samples

**Fig 3:**
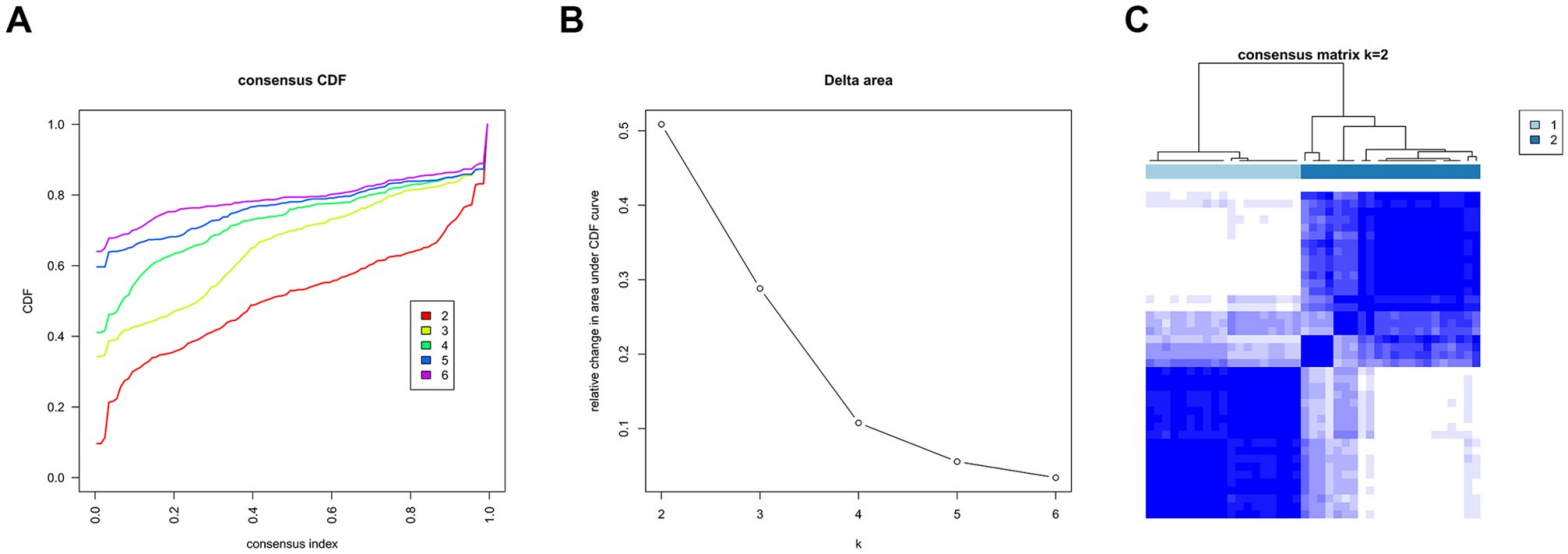
Cluster classification results of diabetic nephropathy samples.

Consistent clustering of diabetic nephropathy samples was conducted based on the expression data of the iron death factor. The cluster started from the number of category k = 2 and the number of category increased one by one until it reached the set maximum (k = 6). The most appropriate number of clusters was chosen, according to results presented in Fig 3A and 3B. A curve with a smaller CDF descending slope was selected in Fig 3A and a higher CDF value was chosen in Fig 3B. In this study, k = 2 is the most appropriate parameter. The heat map for cluster with k = 2 was draw (Fig 3C).

According to the results of consistent clustering, disease samples can be divided into two categories. There are 41 disease samples, among which Group1 contains 19 samples and Group2 contains 22 samples.

### Analysis of differential gene expression in samples of diabetic nephropathy

**Fig 4:**
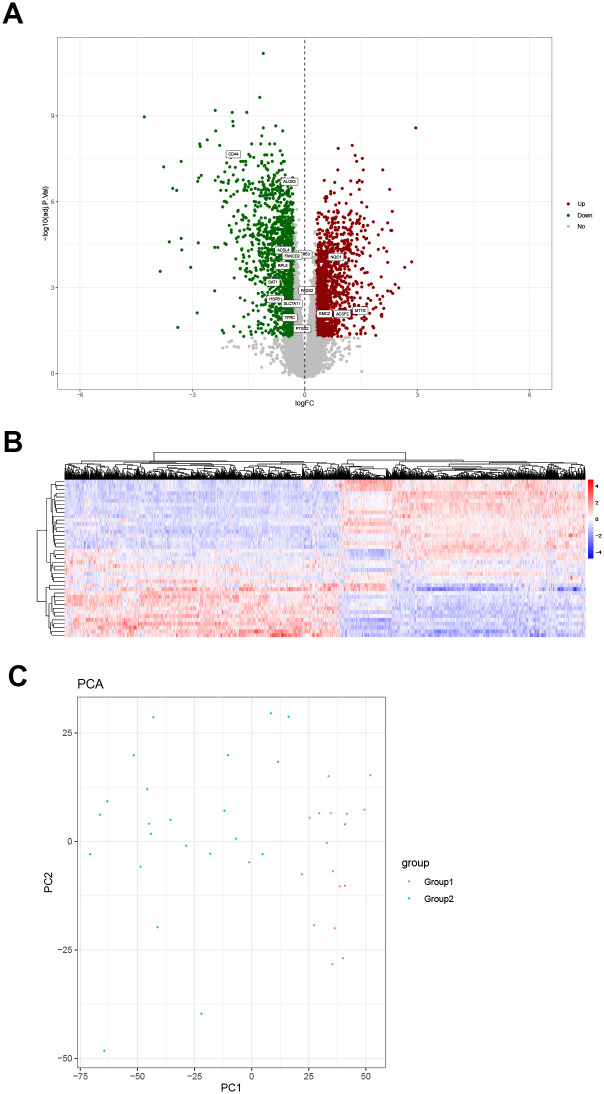
Differential gene expression analysis diagram and principal component analysis. (A) Volcano map. The green dots, down-regulated differential genes; the red dots, up-regulated differential genes; the gray dots, genes that are not differentially expressed. Sixteen of the iron death genes are among the differentially expressed genes. (B) Heat map of differential gene expression. The abscissa represents gene clustering. The more the genes are expressed in the same amount in the sample, the closer they are in the figure. The ordinate represents the clustering of samples. The more the gene expression levels are the same among samples, the closer they are in the picture. The color scale represents the abundance of gene expression. The red presents up-regulation and the blue presents the down-regulation. (C) Principal component analysis based on differentially expressed genes.

Based on the results of disease sample typing, different types of samples were screened for differentially expressed genes. The result was presented as a volcano map (Fig 4A). A total of 3169 differentially expressed genes were screened, of which 1496 genes were upregulated and 1673 were downregulated. Overlapping analysis of differentially expressed genes and iron death genes revealed that 16 of the 60 iron death genes were differentially expressed in diabetic nephropathy, which are shown in Fig 4A. In this study, a total of 30905 gene expression differences were analyzed, and 3169 differentially expressed genes were screened. Among the 60 iron death genes, 16 were differentially expressed in diabetic nephropathy, and the differentially expressed genes were enriched, with an enrichment ratio of 2.6 (16 /60:3169/30905 = 2.6). Among them, CD44, ALOX5, ACSL4, FANCD2, RPL8, SAT1, HSPB1, SLC7A11, TFRC, PTGS2, FADS2, and TP53 are highly expressed in Group1, and NQO1, EMC2, ACSF2, MT1G are relatively lowly expressed in Group1. The expression heat map is shown in Fig 4B.

### Principal component analysis based on differentially expressed genes

In order to test the effect of disease classification, principal component analysis was performed based on the differentially expressed genes between different types of samples, and a bubble chart was drawn (Fig 4C). It can be seen from the figure that there are obvious differences between samples of different types.

### Gene function enrichment analysis

**Fig 5:**
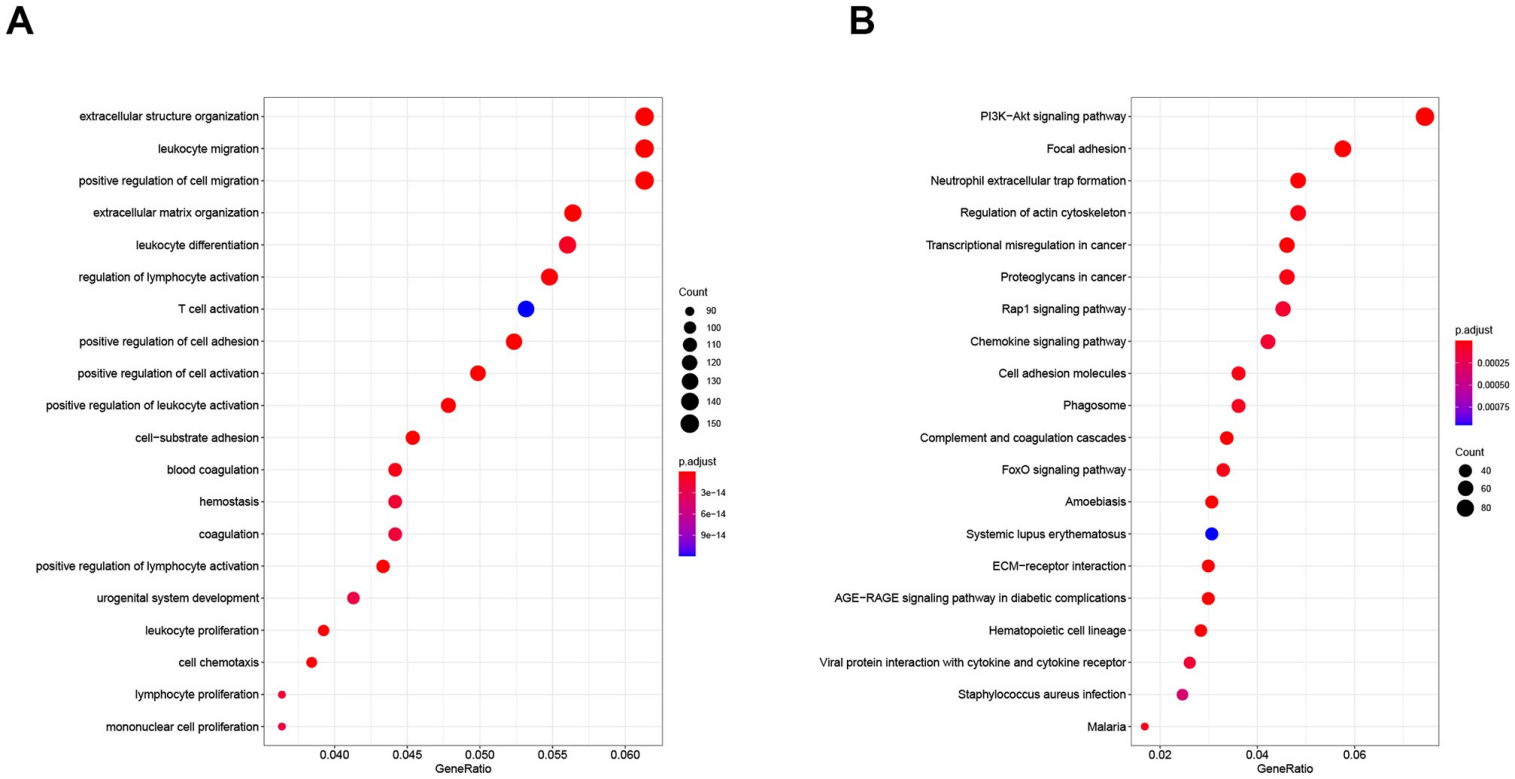
Gene function enrichment analysis: GO function enrichment analysis (A) and KEGG pathway enrichment analysis (B).

The GO function and KEGG pathway enrichment analysis of differentially expressed genes were performed. The GO biological process enrichment analysis yielded 1315 enrichment categories, including cell migration, cell differentiation, extracellular matrix production, cell proliferation, cell activation regulation, and cell chemotaxis, cell-matrix adhesion, coagulation, urogenital system development and other biological processes. KEGG pathway enrichment analysis yields 73 enrichment pathways, including PI3K-Akt signaling pathway, AGE-RAGE signaling pathway, FoxO signaling pathway, Rap1 signaling pathway, Chemokine signaling pathway, ECM-receptor interaction and so on. Taking the top 20 most significant enrichment categories and pathways, respectively and the results were plotted in Fig 5.

### Differential gene co-expression analysis

**Fig 6:**
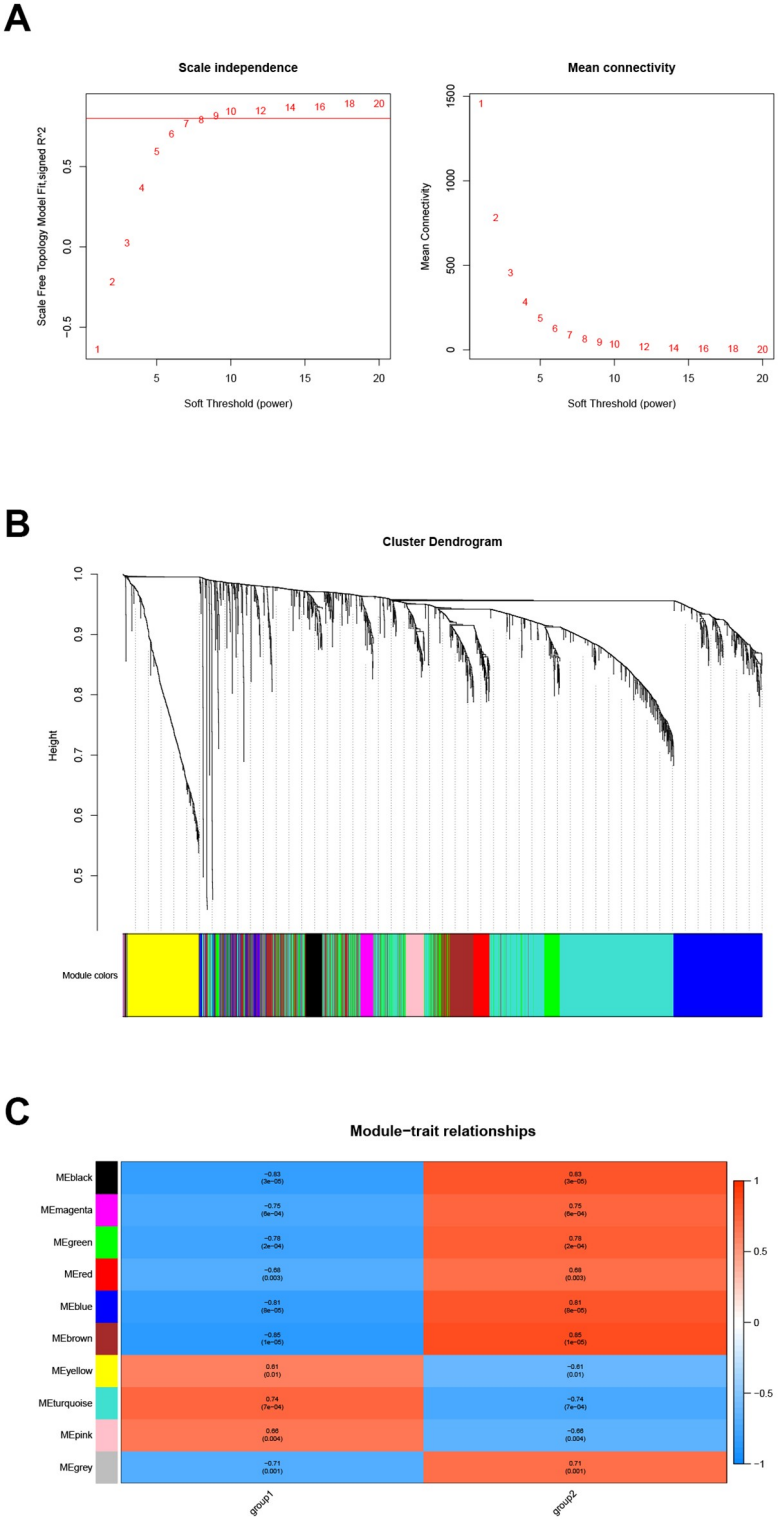
Gene co-expression analysis. Soft threshold screening (A), hierarchical clustering graph (B) and trait association analysis result graph (C).

Extracting the expression data of differentially expressed genes in samples of diabetic nephropathy patients for co-expression analysis. First, the soft threshold is selected for subsequent co-expression network construction (Fig 6A). The principle is to make the constructed network more in line with the characteristics of the scale-free network. The R-square was set as 0.8 (Fig 6A). Using WGCNA to construct the co-expression network module and visual display of the gene correlation in the modules. A total of 9 co-expression modules were obtained. The number of genes in each module is at least 30. The results were displayed in a hierarchical clustering diagram (Fig 6B). The number of module genes is between 76 and 1154, of which 8 genes were not clustered into any module and were marked in gray. Performing trait correlation analysis between the module and the disease sample typing results, and extracting the most relevant module Brown as the key module. Finally, this module contains 464 genes (Fig 6C).

### Hub gene screening

**Fig 7:**
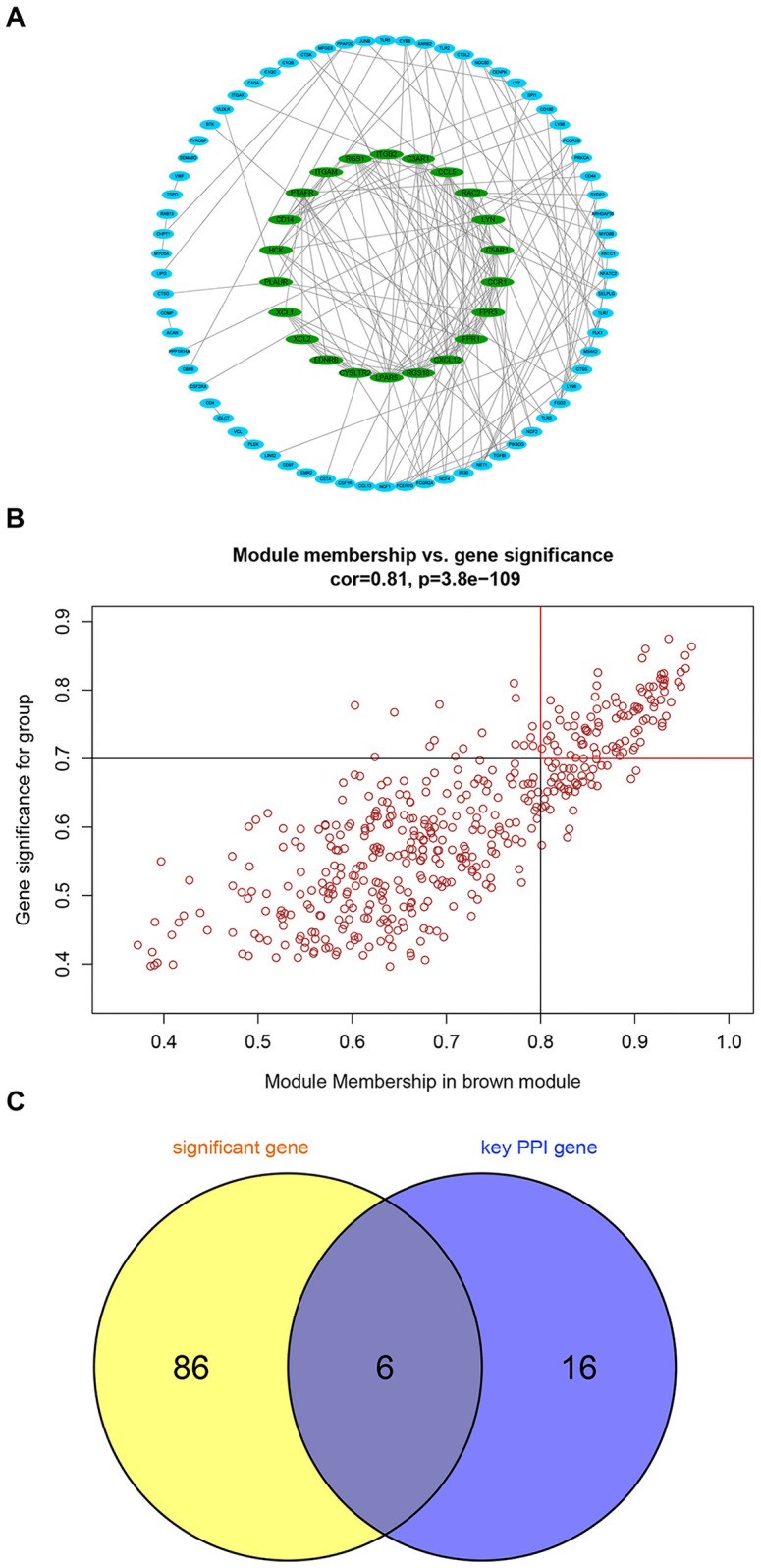
Hub gene screening. Protein interaction screening candidate key genes (A), Modular gene significance screening candidate key genes (B) and the Venn diagram (C).

The human protein interaction database (String) was used to analyze the protein interaction of key module genes. The PPI network diagram is shown in Fig 7A. A total of 177 protein interaction pairs were obtained and 22 genes with protein interaction number ≥ 6 were selected as candidate key genes. At the same time, the candidate key genes were screened by module gene significance, and 92 genes with Module Membership ≥ 0.8 and Gene significance ≥ 0.7 were screened as candidate key genes (Fig 7B). Taking the intersection of two sets of candidate key genes and drawing a Venn diagram, and the 6 genes (FPR3, C3AR1, CD14, ITGB2, RAC2, and ITGAM) with overlapping candidate key genes was used as Hub genes (Fig 7C).

### Hub gene correlation analysis and expression differences in different types

**Fig 8:**
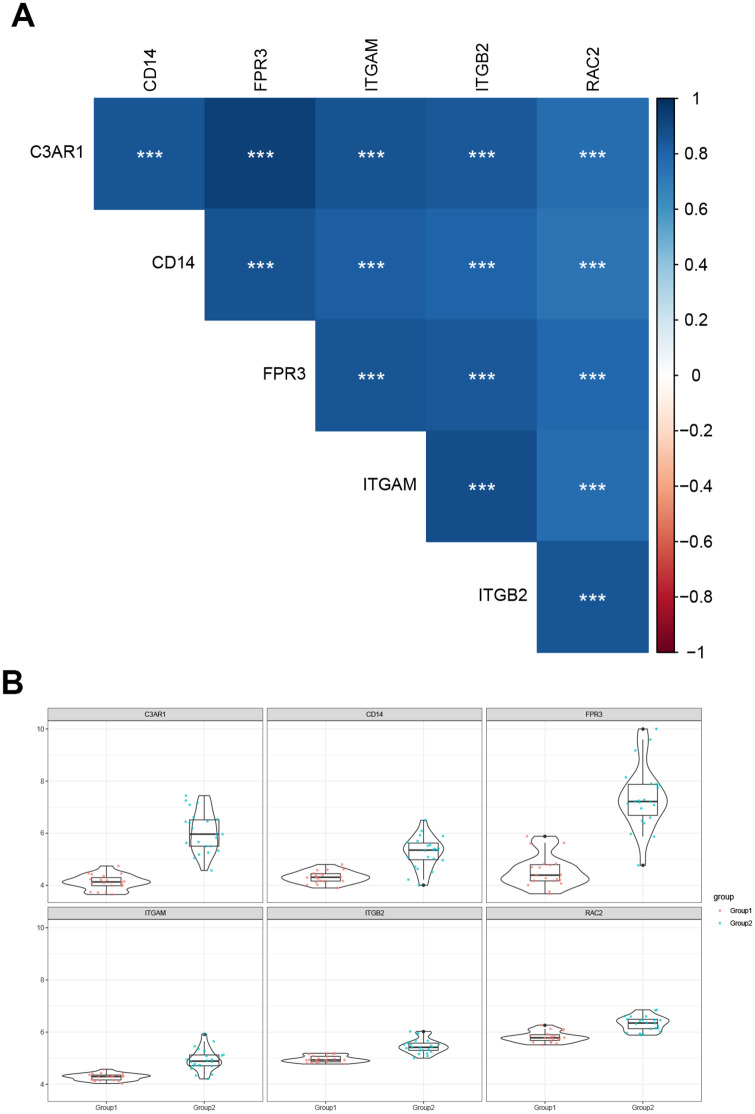
Hub gene correlation analysis and expression differences in different types. Hub gene correlation analysis (A). Differential analysis of Hub gene expression in different types (B).

The expression data of Hub gene in diabetic nephropathy samples were extracted for correlation analysis, and the results showed that the expression of Hub genes were highly correlated (Fig 8A). At the same time, the expression level comparison and difference analysis were performed, and the results showed that the expression levels of all Hub genes in Group2 were higher than those in Group1 (Fig 8B).

### Hub gene multi-factor regulatory network

**Fig 9:**
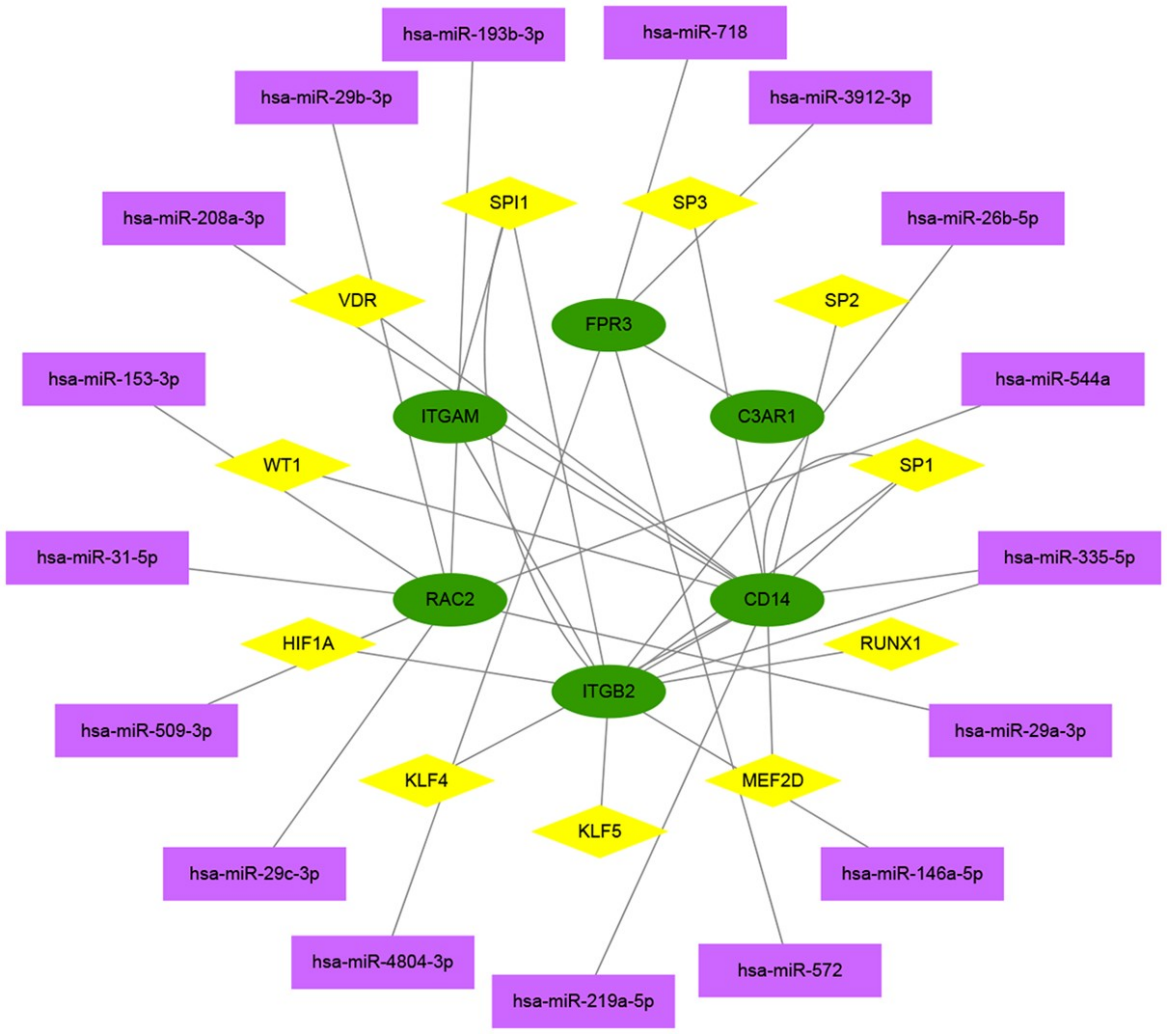
Hub gene multi-factor regulatory network. Colors and shapes indicate different factor types. Green is hub gene mRNA, pink is transcription factor, and purple is non-coding gene miRNA.

TF and ncRNA play important roles in regulating the expression and function of protein-coding genes. Using principal component analysis to find out the main regulators that interact with the Hub gene. After principal pivot analysis, 17 main regulatory ncRNAs were obtained, all of which were miRNAs, and 11 main regulatory TFs were obtained (Fig 9). Analyzing the functions of miRNA and TF in previous studies, it was found that the non-coding genes hsa-miR-572 [21], hsa-miR-29a-3p [22], hsa-miR-29b-3p [23], hsa -miR-208a-3p [24], hsa-miR-153-3p [25], hsa-miR-29c-3p [26] may be related to diabetic nephropathy and transcription factors HIF1α [27], KLF4 [28], KLF5 [29], RUNX1 [30], SP1 [31], VDR [32], WT1 [33] may be related to diabetic nephropathy.

## Discussion

Diabetic nephropathy is one of the most common microvascular complications of diabetes. It is characterized by proteinuria and progressive decline in renal function. The pathological features include the deposition of extracellular matrix in the glomerulus and tubular interstitium, and the death of glomerulus and renal tubule cells [34], which eventually leads to end stage renal disease (ESRD). Diabetic nephropathy accounts for approximately 16.4% of ESRD cases in China [35]. Therefore, it is important to clarify the pathogenesis of diabetic nephropathy and formulate therapeutic interventions.

Iron death is a newly discovered form of cell death in recent years. It is characterized by lipid hydroperoxides accumulation induced cell death, which is different from apoptosis, necrosis and autophagy on the phenotypes of morphology, biochemistry and genetics [36]. Previous studies have reported that iron death is related to various kidney diseases such as polycystic kidney disease, acute kidney injury and renal clear cell carcinoma [37–40]. However, its regulatory role in diabetic nephropathy is still unclear.

This study is based on the iron death gene to classify diabetic nephropathy samples, which is helpful to analyze the heterogeneity of diabetic nephropathy. Our result showed that the expression of iron death factor was different between the diabetic nephropathy group and the normal control group. Based on the expression data of the iron death factor, consistent cluster analysis of disease samples was conducted and the disease samples can be divided into two categories. Principal component analysis showed that the two types are significantly different. Gene differential expression analysis showed that 16 of the 60 iron death genes were differentially expressed and were enriched among differentially expressed genes. Based on the differential genes, the key disease modules were screened. Finally six Hub genes related to iron death in diabetic nephropathy were obtained, including FPR3, C3AR1, CD14, ITGB2, RAC2 and ITGAM.

Formylpeptide Receptor (FPR) is a G protein-coupled receptor [41]. Activation of FPR3 regulates cell proliferation, apoptosis and angiogenesis, and may also trigger signal cascade reaction through ligand-receptor binding on immune cells, which leads to new gene transcription, mediator release or cell migration [42]. Inflammation mediates the angiogenesis of proloferative diabetic retinopathy (PDR), and FPR antagonists can inhibit the inflammation and neovascularization caused by the PDR vitreous body [43]. There is no research to clarify the role of FPR3 in inflammation and immunity of diabetic nephropathy, but the intervention of FPR3 may become a new treatment strategy for diabetic nephropathy. As the receptor of complement C3a, C3AR1 is a key mediator of inflammation and participates in cellular inflammatory response [44]. Studies have found that the synthesis of complement C3 in renal tubular epithelial cells of mice with unilateral ureteral obstruction increases, and the expression of complement receptor C3AR1 in mesenchymal cells increases [45]. Moreover, the expression of C3AR1 in kidney tissue is closely related to the severity of kidney injury [46]. Based on the results of previous studies and this study, it is speculated that C3AR1 can be used as a target for both diagnosis and treatment. CD14 is a high-affinity lipopolysaccharide (LPS) receptor that binds to Toll-like family receptors to activate the immune response induced by LPS [46]. CD14 also can induce interleukin (IL), tumor necrosis factor -α (Tumor Necrosis Factor-α, TNF-α) and other related inflammatory factors and chemokine expression, which ultimately effectively regulate cell proliferation, transformation and apoptosis [47]. CD14 is expressed in proximal tubular epithelial cells and distal nephron epithelial cells in kidney tissue [48]. Studies have found that the number of circulating CD14 monocytes in patients with kidney disease is significantly reduced [49]. The number of CD14 monocytes is negatively correlated with the severity of the disease and positively correlated with renal function [49].

Type 2 diabetic nephropathy patients have pro-inflammatory CD14^+^ and CD16^+^ monocyte disorder [50]. This abnormal immune function may be related to the activation of nuclear factor kappa-B (NF-κB)/Toll-Like Receptor 4 (TLR4) inflammatory signaling pathways [50]. Based on the above studies, it is speculated that the chronic immune inflammatory response, mediated by CD14, may be involved in the progression of diabetic nephropathy. ITGAM and ITGB2 belong to the integrin family. Integrin β2 (ITGB2), namely CD18, can bind to the adhesion molecules on the surface of endothelial cells (such as intercellular cell adhesion molecule-1 (ICAM-1)) to mediate the interaction between leukocytes and endothelial cells. And its cytoplasmic region can be connected with a variety of cytoskeleton proteins to participate in signal transduction [51]. ITGAM encodes the α chain of integrin αMβ2 (CD11b). CD11b can form leukocyte adhesion molecule β2 integrin with CD18, namely macrophage differentiation antigen-1 (Mac-1) [52]. The expression of Mac-1 and ICIAM-1 in the proliferative diabetic retina indicates that adhesion molecules play a role in the pathogenesis of diabetic microvascular complications [53]. Ras-related C3 botulinum toxin substrate (Ras-related C3 botulinum toxin substrate, Rac) is a small molecule Rho-GTPase and it plays an important role in regulating cell proliferation, sugar metabolism, cell motility, superoxide production, cellular immunity and other biological processes [54, 55]. Studies have found that RAC2 can be used as a biomarker for the prognosis of renal clear cell carcinoma and promote the progression of renal clear cell carcinoma [56]. However, its role in the pathogenesis of diabetic nephropathy remains to be studied.

The above-mentioned key genes related to iron death in diabetic nephropathy are mainly related to the immunity and inflammation of diabetic nephropathy. Chronic immune inflammation occupies an important position in the pathogenesis of diabetic nephropathy. And there is a parallel relationship between the degree of immune inflammation and the degree of kidney damage [57]. A variety of inflammatory factors and chemokines recruit and activate the local microenvironmental immune status of the kidney, mediate pathological changes such as glomerular stromal proliferation and basement membrane thickening and damage the normal structure and function of the glomerulus, which finally promotes the occurrence and development of diabetic nephropathy [58].

The post-transcriptional regulation of protein-coding genes by ncRNAs has always been thought to be related to the occurrence of diabetic nephropathy, but mechanism of its regulation role on diabetic nephropathy-related genes is still unclear. In this study, Pivot analysis was used to further conclude that the main regulators interacting with the Hub genes are transcription factors and miRNAs. According to the analysis of published literature, the non-coding genes hsa-miR-572, hsa-miR-29a-3p, hsa-miR-29b-3p, hsa-miR-208a-3p, hsa-miR-153-3p and hsa-miR-29c-3p may be related to diabetic nephropathy. And transcription factors HIF1α, KLF4, KLF5, RUNX1, SP1, VDR, WT1 may be also related to diabetic nephropathy. The above factors and Hub genes are collectively regulated and involved in the occurrence and development of diabetic nephropathy. Moreover, these factors and genes may be potential target for therapeutic drugs.

When the human genome is transcribed, many regulatory non-coding RNAs (ncRNAs) are produced, including MicroRNAs, LncRNAs, circRNAs, etc. ncRNAs regulate gene expression at different physiological levels and affect the epigenetic characteristics of kidney disease [59]. With the development of computational biology and sequencing technology, a large number of ncRNAs have been discovered, which play an important role in many biological activities such as epigenetic regulation. With the increase in the amount of biological sequencing data, predicting the association between non-coding RNA and disease through bioinformatics analysis will help provide directions for further biological experiments [60]. This study uses systems biology methods, combined with WGCNA, crossover genes, and module analysis-based functions and pathways to determine the biological processes and signal pathways related to iron death in diabetic nephropathy, as well as genes and molecular networks related to iron death in DN. This study will provide new possibilities for understanding the molecular mechanism of diabetic nephropathy and provide a basis for subsequent research on the correlation between diabetic nephropathy and iron death.

This study analyzed the biomarkers of iron death genes in diabetic nephropathy based on bioinformatics technology. This research promotes the understanding of the pathogenesis of diabetic nephropathy at the RNA level and the development of potential drug targets for clinical treatment, which ultimately provides guidance for disease diagnosis, treatment, prognosis and prevention. Computational models have become an important means of identifying new RNA-disease associations. Establishing a computational model to predict and quantify the association between human non-coding RNA and disease can effectively find the most relevant RNA-disease for experimental verification and reduce the time and cost of biological experiments [61]. In addition, computational models can also be used to predict the potential functions of non-coding RNAs, identify new genes, and construct potential regulatory networks between non-coding RNAs and other molecules at different levels [61]. Many computational models have been applied to bioinformatics research, such as LncRNA-miRNA interaction prediction [60, 62, 63], LncRNA-disease association prediction [64], miRNA-disease association prediction [65, 66] and circRNA-disease association prediction [67]. In the future, it is a hot topic worthy of further research that the establishment of effective computational models to integrate different biological information and to make full use of different types of data sources to systematically study the relationship between circRNAs, miRNAs, LncRNAs and human diseases.

## Materials and methods

### Data collection and preprocessing

Gene expression data related to diabetic nephropathy was obtained from RNA-seq database (https://www.ncbi.nlm.nih.gov/geo/query/acc.cgi?acc=GSE96804). Ferroptosis genes were selected from previous report [19].

### The expression distribution of iron death factor in the diabetic nephropathy

Extracting the expression data of iron death genes from the GSE96804 data set and using the Limma package for differential expression analysis. FC stands for fold change, which represents the ratio of expression levels between the two sets of samples, and log base 2 of FC is log_2_FC. FDR stands for False Discovery Rate, which is obtained by correcting the p-value of the significance of the difference. In the differential expression analysis, the Benjamini-Hochberg correction method was used to correct the significance p-value obtained from the hypothesis test during transcriptome sequencing, and finally FDR was used as the key indicator for the screening of differentially expressed genes. Generally, FDR<0.01 or 0.05 is taken as the default standard. In this study, FDR<0.05 and |log_2_FC|>0.3 were set as the threshold to screen high-confidence differentially expressed genes. Differential expression analysis was performed by sample type (diabetic nephropathy group and normal control group) and gender (male and female).

### Typing of diabetic nephropathy samples

Using the ConsensusClusterPlus package to perform consistent clustering of disease samples based on the iron death factor expression data. Cluster analysis was initiated with the cluster number set to 2 and then the number of cluster was increased one by one until the specified maximum number of category was reached. Finally, evaluating and selecting the most suitable number of cluster and then drawing a heat map of the expression model.

### Differential gene expression analysis

Based on the typing results of diabetic nephropathy samples, different types of samples were analyzed for differential gene expression. The differentially expressed genes were screened using the Limma package, with the parameters of FDR<0.05 and |log_2_FC|>0.3. Then, the volcano maps and expression heat maps were drawn. Finally, the difference gene and the iron death gene were overlapped and analyzed.

### Principal component analysis based on differentially expressed genes

In order to test the effect of diabetic nephropathy classification, principal component analysis was performed based on the differentially expressed genes between different types of samples.

### Gene function enrichment analysis

Using R clusterProfiler, with FDR<0.05 as the screening threshold, GO biological process and KEGG pathway enrichment analysis were performed on the above differentially expressed genes. Both GO BP analysis and KEGG pathway enrichment analysis were conducted using the clusterProfiler package in R, and p<0.05 was used as the threshold to screen the significantly enriched GO and KEGG categories. Finally, top 20 most significant gene functions were selected and plotted.

### Gene co-expression analysis

Extracting the expression data of the differentially expressed genes in the samples of diabetic nephropathy patients and using the WGCNA algorithm for co-expression analysis. Weighted Correlation Network Analysis (WGCNA, Weighted correlation network analysis) is a systems biology method used to describe gene correlation patterns between different samples. This method can be used to identify highly coordinated gene sets, and identify candidate biomarker genes or therapeutic targets based on the interconnectivity of the gene sets and the association between gene sets and phenotypes. Weighting refers to the power operation of the correlation value, and the power value is the soft threshold [68]. First, the soft threshold is selected. The principle is to make the constructed network more in line with the characteristics of the scale-free network. Setting R-square = 0.8 and the number of genes in each module is at least 30. Moreover, the results are displayed in a hierarchical clustering tree. Performing trait correlation analysis between the module and the disease sample typing results, and extracting the most relevant module Brown as the key module.

### Hub gene screening

Using the human protein interaction database (String) to analyze the protein interaction of key module genes. Screening the interactions with a comprehensive score> 900 to obtain protein-protein interactions, and screening genes with protein interactions ≥ 6 as candidate key genes. Furthermore, using Cytoscape to draw PPI network diagram. Meanwhile, candidate key genes were screened by module gene significance, and the genes with Module Membership≥0.8 and Gene significance≥0.7 were used as candidate key genes. Finally, the overlapping part of the two sets of candidate key genes is regarded as the Hub genes.

### Correlation analysis and expression differences in different types of Hub genes

First, extracting the expression data of Hub genes in disease samples and performing correlation analysis. Secondly, calculating correlation coefficient and significance and drawing analysis graph. Finally, performing expression comparison and difference analysis.

### Hub gene multi-factor regulatory network

Using RAID v2.0 (www.rna-society.org/raid2/) database to predict non-coding gene-gene (protein) interaction. Using TRRUST V2 (www.grnpedia.org/trrust) database to predict transcription factor- Gene (protein) interaction. Using principal component analysis method, based on TRRUST V2 database and RAID v2.0 database to find out the main regulatory factors that interact with the Hub gene (p<0.05). Using Cytoscape to plot the results.
